# Layer‐by‐Layer Multidermal Rejuvenation

**DOI:** 10.1111/jocd.71004

**Published:** 2026-07-01

**Authors:** Kyu‐Ho Yi, Suyeon Lee, Ting Song Lim, Diala Haykal

**Affiliations:** ^1^ You and I Clinic Seoul Republic of Korea; ^2^ Medical Research Inc. Wonju Republic of Korea; ^3^ Clique Clinic Kuala Lumpur Malaysia; ^4^ Centre Laser Palaiseau Palaiseau France

**Keywords:** rejuvenation, skin aging, skin boosters, skin quality

## 1 Introduction

The clinical label “skin rejuvenation” encompasses multiple patient priorities, including smoother texture, brighter tone, more uniform pigmentation, reduction of fine lines, improved firmness, and enhanced facial contour. These clinical domains arise from distinct anatomical and biological substrates rather than from a single structural layer. Chronological aging and photoaging alter epidermal turnover and pigmentation, remodel collagen and elastin throughout the dermis, and modify the mechanical support provided by superficial fat compartments and the fibroseptal network [1]. Therefore, facial skin‐quality optimization should be understood as a multidimensional and depth‐dependent process rather than as a single‐surface problem.

**FIGURE 1:**
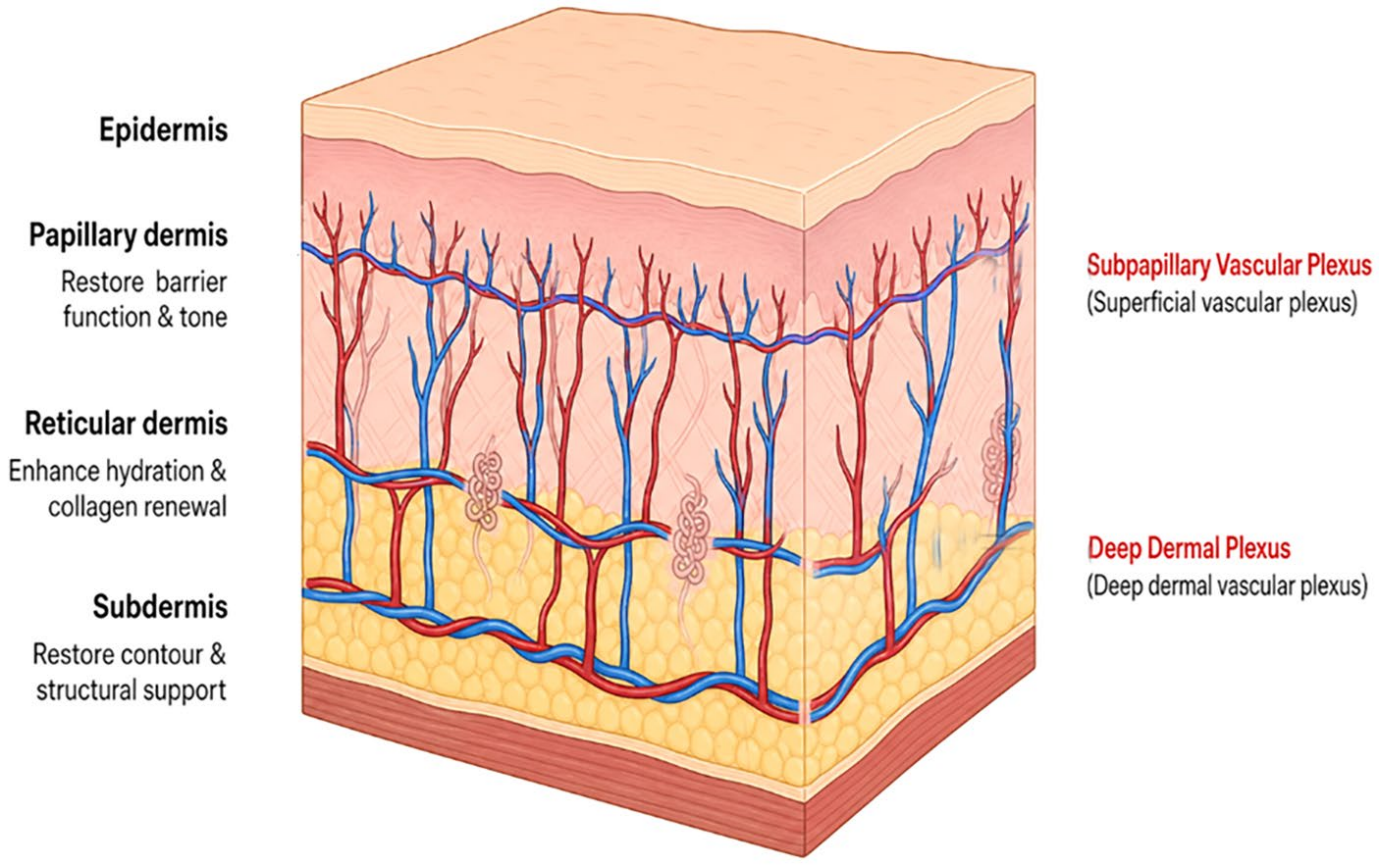
Anatomical organization of the skin and functional targets for layer‐specific rejuvenation. Layered organization of the skin and depth‐dependent functional targets relevant to multidermal rejuvenation. The epidermis, papillary dermis, reticular dermis, and subdermis are shown together with the subpapillary vascular plexus and deep dermal plexus. Each layer contributes to distinct aspects of skin quality, including barrier function, pigmentation, and optical texture at the epidermal level; hydration, superficial turgor, and early fine lines at the papillary dermal level; collagen remodeling, elasticity, and firmness at the reticular dermal level; and structural support, laxity, and contour at the subdermal level.

Because these processes occur across multiple anatomical depths, treatment strategies that focus on a single modality may inadequately address the dominant driver of visible skin aging. In clinical practice, when depth‐dependent contributors are not explicitly considered, clinicians may compensate by increasing treatment energy, repeating procedures, or empirically combining products and devices. Such approaches may increase inflammation, downtime, cost, and complication risk without proportionally improving outcomes. This limitation is particularly relevant because many contemporary rejuvenation strategies remain organized around treatment categories—such as injectables, energy‐based devices, resurfacing, or topical regimens—rather than around the anatomical substrates responsible for the patient's primary concern. An anatomically informed strategy may therefore provide a more coherent basis for treatment planning. Recognizing that different skin‐quality concerns originate predominantly from different tissue layers allows clinicians to align interventions with the anatomical depth most responsible for the observed changes (Figure 1). We therefore propose a practical anatomy‐guided framework for layer‐by‐layer multidermal rejuvenation. The framework involves three steps: (1) identifying the skin‐quality domains most relevant to the patient, (2) mapping each domain to its dominant anatomical contributors—epidermis, papillary dermis, reticular dermis, or subdermis—and (3) selecting and sequencing interventions that primarily act at those depths (Figure 1).

The importance of this narrative lies in reframing facial rejuvenation from a product‐centered or procedure‐centered approach into a layer‐specific anatomical logic. Its novelty is not the introduction of a single new device, injectable, or topical agent, but the integration of established and emerging modalities into a unified depth‐based framework. This framework is not intended to replace clinical judgment or device‐specific treatment protocols. Rather, it provides a consistent anatomical rationale for modality selection, expectation setting, procedural sequencing, risk reduction, and the construction of staged multimodal treatment plans that are biologically coherent and risk‐adapted.

## 2 What Is New in the Layer‐by‐Layer Multidermal Framework?

The new contribution of this narrative is the organization of facial skin‐quality optimization according to anatomical layer and functional tissue target rather than by treatment modality alone. Conventional rejuvenation discussions frequently group interventions as topical therapy, resurfacing, injectables, skin boosters, biostimulators, or energy‐based devices. Although clinically useful, such modality‐based categories do not always explain why a treatment should be selected for a specific patient concern, why treatments should be sequenced in a particular order, or why one layer should be prioritized over another.

The proposed multidermal framework instead begins with the anatomical origin of the clinical problem. Epidermal changes are linked to barrier dysfunction, dyschromia, and optical texture; papillary dermal changes are linked to hydration, fine lines, and superficial turgor; reticular dermal changes are linked to collagen remodeling, elasticity, and firmness; and subdermal changes are linked to support, contour, and deeper laxity. This structure allows clinicians to ask a different planning question: not simply “Which treatment should be used?” but “Which anatomical layer is the dominant contributor to the patient's concern, and which intervention best targets that layer with the lowest appropriate procedural burden?”

This framework is also new in its emphasis on sequencing and risk adaptation. Rather than combining procedures empirically, layer‐specific planning encourages stabilization of the epidermal barrier and pigmentary risk before more inflammatory procedures, followed by dermal remodeling and subdermal support when indicated. This may be especially relevant for patients with darker phototypes or inflammatory sensitivity, in whom cumulative procedural inflammation can increase the risk of postinflammatory hyperpigmentation. In this way, the framework links aesthetic outcome, anatomical precision, procedural safety, and treatment equity within a single planning model (Table 1).

**TABLE 1:** Anatomy‐guided layer‐by‐layer framework for facial skin‐quality optimization.

Anatomical layer	Dominant clinical domains	Primary biological substrate	Treatment objective	Framework contribution
Epidermis	Dyschromia, roughness, dullness, barrier instability	Keratinocyte turnover, melanogenesis, stratum corneum integrity	Improve barrier function, pigment balance, and optical texture	Establishes the safety foundation before more inflammatory procedures
Papillary dermis	Fine lines, superficial dehydration, reduced turgor	Superficial collagen, hydration, microvasculature, dermoepidermal junction	Improve hydration, superficial remodeling, and early texture change	Balances visible refinement with risk of superficial inflammation
Reticular dermis	Firmness loss, elasticity decline, deeper rhytids	Collagen bundles, elastin architecture, extracellular matrix remodeling	Promote structural remodeling and neocollagenesis	Distinguishes delayed remodeling from immediate volumization
Subdermis and fibroseptal network	Laxity, shadowing, contour change, support loss	Superficial fat compartments, fibroseptal network, mechanical support	Restore support, contour, and deeper tightening	Recognizes that some “skin‐quality” concerns originate below the dermis

### 2.1 Layer 1: Epidermis—Barrier, Pigment Balance, and Optical Texture

#### 2.1.1 Dominant Aging Change

The epidermis is the interface between skin and environment. Aging and ultraviolet exposure can impair barrier function, slow keratinocyte turnover, and dysregulate melanogenesis [2]. Clinically, this presents as rough texture, diminished radiance, and mottled pigmentation. In richly pigmented skin, procedure‐induced inflammation can trigger postinflammatory hyperpigmentation (PIH), making epidermal optimization a safety step as well as an aesthetic one [3, 4].

#### 2.1.2 Layer‐Matched Interventions

Foundational measures include daily photoprotection, gentle cleansing, and barrier repair with humectants and ceramide‐rich moisturizers. Evidence‐supported topicals—particularly retinoids—improve fine wrinkling and dyschromia over time and may enhance epidermal differentiation and dermal matrix signaling [5]. When procedural epidermal interventions are used, the priority is controlled superficial remodeling with minimal thermal injury. Examples include light chemical peels and conservative nonablative fractional resurfacing, supported by pre‐ and post‐procedure regimens that preserve barrier integrity [6]. In patients prone to PIH, conservative densities and adequate intervals may reduce pigmentary sequelae.

Within the proposed framework, epidermal optimization is important because it functions as both an aesthetic target and a safety gatekeeper before deeper or more inflammatory interventions.

### 2.2 Layer 2: Papillary Dermis—Hydration, Microvasculature, and Early Collagen Loss

#### 2.2.1 Dominant Aging Changes

The papillary dermis supports the dermoepidermal junction and contributes substantially to skin “turgor.” Early aging involves reduced hydration and progressive collagen changes that reduce recoil and accentuate fine lines. This layer is capillary dense and richly innervated, which helps explain why some superficial dermal interventions are associated with more pain, bruising, and visible inflammation.

#### 2.2.2 Layer‐Matched Interventions

Three modality families commonly act at this depth: superficial energy‐based devices, microneedling‐based approaches, and intradermal injectables. Nonablative fractional lasers and superficial radiofrequency (RF) microneedling create controlled micro‐injury intended to stimulate remodeling while preserving most of the epidermis [7]. RF systems are often described as less dependent on epidermal chromophores than lasers, which is relevant for darker phototypes; nevertheless, technique and parameter selection remain critical. In 2025, the U.S. FDA issued a safety communication describing potential risks when RF microneedling devices are used in ways that exceed cleared indications or by inadequately trained users, highlighting burns, scarring, pigmentary change, infection, and fat loss as potential adverse outcomes [8].

Intradermal hyaluronic acid (HA) products are widely referred to as “skin boosters,” yet definitions and classifications vary by region and manufacturer [9]. Clinically, intradermal HA is typically delivered in small aliquots to improve hydration and fine texture. The injection microenvironment matters: intradermal placement traverses a neurovascular‐rich plane and may be associated with greater pain and more visible inflammation. When minimal downtime is a priority, clinicians may consider approaches that influence dermal quality with less superficial trauma (e.g., conservative energy‐based settings or deeper planes when appropriate).

In the multidermal framework, the papillary dermis represents the layer in which visible fine‐texture improvement must be balanced against the greater likelihood of pain, bruising, erythema, and pigmentary sequelae associated with superficial dermal intervention.

### 2.3 Layer 3: Reticular Dermis—Tensile Strength and Structural Remodeling

#### 2.3.1 Dominant Aging Changes

The reticular dermis provides tensile strength through thicker collagen bundles and elastic fiber architecture. Aging disrupts collagen organization and can promote maladaptive remodeling that contributes to laxity and deeper rhytids. Interventions intended to improve firmness and structural skin quality often need to reach the mid‐to‐deep dermis.

#### 2.3.2 Layer‐Matched Interventions

Higher‐energy nonablative fractional devices and, in selected patients, ablative fractional resurfacing can induce controlled remodeling in the reticular dermis. In darker phototypes, the risk of prolonged erythema and dyspigmentation warrants conservative settings, test‐spotting when appropriate, and longer intervals between sessions.

Biostimulatory injectables provide another reticular‐dermal strategy by promoting neocollagenesis through controlled wound‐healing responses. Hyperdiluted calcium hydroxylapatite (CaHA) and poly‐L‐lactic acid (PLLA) are commonly used to improve firmness and skin quality over time, and expert consensus guidance exists regarding dilution concepts and safety considerations [10]. These agents should be understood as remodeling treatments rather than immediate volumizers: clinical benefit is delayed, depends on host response, and is influenced by depth of placement and product distribution.

This distinction is central to the proposed framework because reticular dermal interventions should be evaluated by delayed matrix remodeling and firmness rather than by immediate volume correction alone.

### 2.4 Layer 4: Subdermis and Fibroseptal Network—Support, Contour, and Deep Tightening

#### 2.4.1 Dominant Aging Changes

Below the dermis, superficial fat compartments and the fibroseptal network contribute to contour, glide, and mechanical support. Age‐related changes in fat volume and quality, together with septal and ligamentous alterations, can produce sagging and shadowing that cannot be corrected by resurfacing alone. Addressing this layer is therefore essential for patients whose primary concerns include contour, jawline definition, and laxity.

#### 2.4.2 Layer‐Matched Interventions

Subdermal strategies include structural filler placement, subdermal biostimulation, and energy‐based tightening that targets deeper fibrous layers. A conceptual advantage of subdermal placement is the injection environment: below the subpapillary plexus, vascular and sensory density may be lower than in the superficial dermis, potentially reducing bruising and discomfort while still influencing the overlying dermis through trophic and mechanobiologic signaling. Ultrasound‐evaluated clinical data have reported skin‐quality improvement following subdermal injection of non‐crosslinked HA‐based products, supporting the concept that meaningful dermal change is not exclusive to intradermal delivery [11].

Deep tightening devices, such as microfocused ultrasound with visualization (MFU‐V) and monopolar RF, deliver energy to defined depths to induce collagen contraction and subsequent remodeling in deep dermal and fibromuscular layers [12]. These modalities are best conceptualized as “scaffold modifiers” that complement epidermal and dermal‐focused interventions rather than replacing them.

The inclusion of the subdermis is a key feature of the multidermal framework because some concerns perceived by patients as poor “skin quality,” such as laxity, shadowing, or loss of jawline definition, may originate from deeper support failure rather than from epidermal or dermal surface change alone.

## 3 Sequencing and Synergy: A Staged, Inflammation‐Aware Approach

**FIGURE 2:**
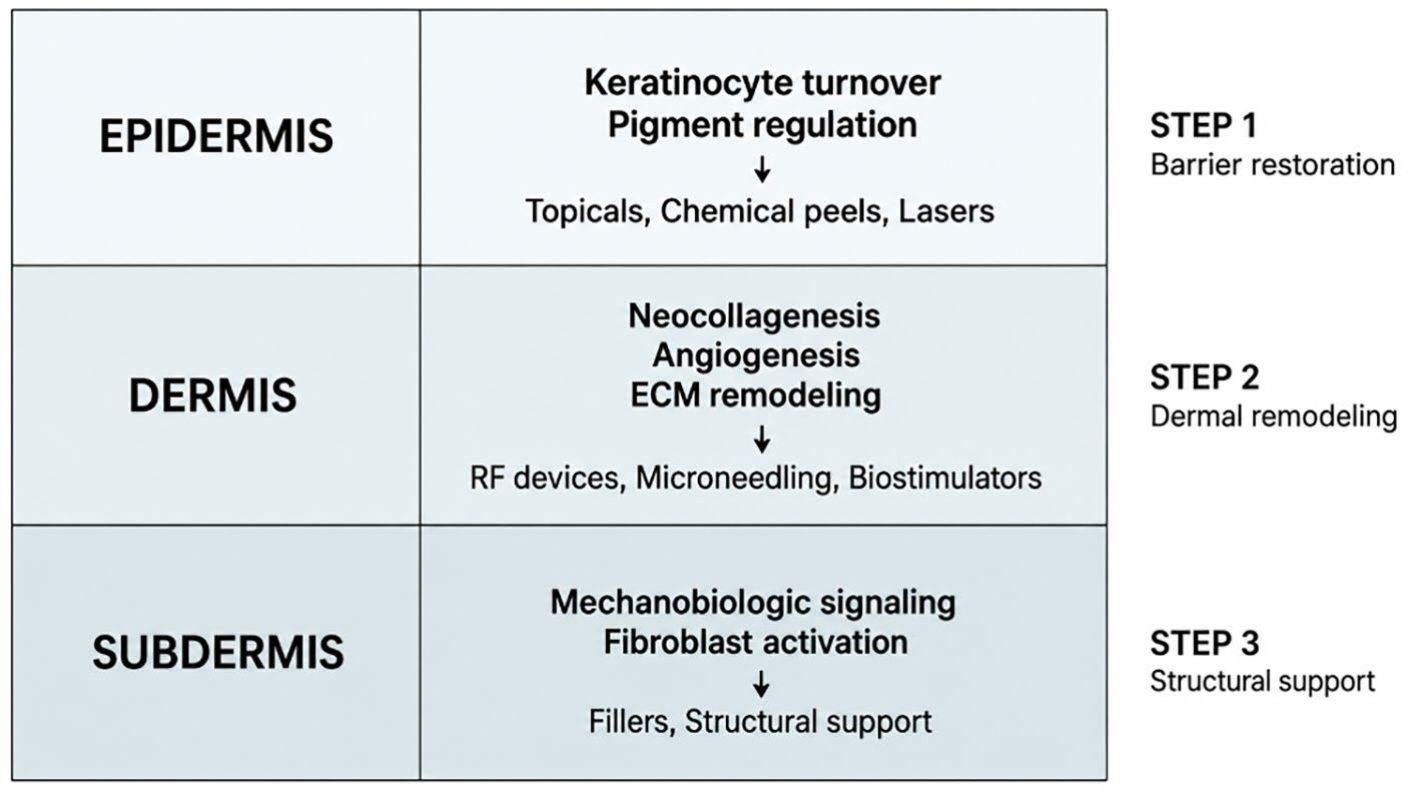
Conceptual framework for layer‐specific multidermal rejuvenation. The framework begins with identification of the patient's dominant skin‐quality concern and maps that concern to the most relevant anatomical layer. Epidermal interventions address barrier restoration, pigmentation, and optical texture. Papillary dermal interventions target hydration, superficial turgor, and fine lines. Reticular dermal interventions promote matrix remodeling, collagen renewal, elasticity, and firmness. Subdermal interventions address support, contour, laxity, and deeper tissue scaffolding. This model emphasizes that multimodal rejuvenation should be defined by layer‐specific purpose and sequencing rather than by the number of procedures performed.

Layered rejuvenation is most useful when it informs sequencing rather than simply adding more procedures (Figure 2). A pragmatic strategy is to stabilize epidermal barrier function and pigment regulation first, then address papillary‐dermal hydration and superficial remodeling, followed by reticular dermal remodeling or biostimulation, and finally subdermal support or tightening when indicated. This progression aligns with wound‐healing biology and helps limit cumulative inflammatory burden.

The novelty of this sequencing model is that combination treatment is not defined by the number of modalities used, but by whether each modality has a distinct anatomical purpose. For example, an epidermal intervention may improve tone and optical texture, a papillary dermal intervention may improve hydration and fine lines, a reticular dermal intervention may improve firmness, and a subdermal intervention may improve support and contour. Interval planning should be individualized based on phototype, baseline sensitivity, comorbid inflammatory dermatoses, and the intensity of each intervention.

## 4 Safety: Anatomy, Imaging, and Complication Preparedness

A depth‐based framework is inseparable from safety. The most severe injectable complications are vascular, including ischemia and rare vision‐threatening events [13, 14, 15]. Reviews and consensus recommendations emphasize meticulous anatomical knowledge, conservative technique, and rapid recognition and management of ischemic signs [13, 14, 15, 16]. Depth mapping itself is a safety tool: attempting to treat deep laxity with aggressive epidermal injury may increase risk without addressing the dominant anatomical driver, while placing product too superficially may increase risks of discoloration, nodules, and inadvertent engagement of the superficial vascular plexus.

High frequency ultrasound can support both prevention and management. Ultrasound is increasingly used to identify existing filler, assess tissue planes in previously treated regions, and evaluate nodules or other complications. When complications occur, ultrasound may help differentiate filler deposition, granuloma, and fibrosis, enabling more targeted management and reducing unnecessary interventions. In this context, the importance of the multidermal framework extends beyond aesthetic planning: it provides a safety‐oriented rationale for matching intervention depth to anatomical target, avoiding unnecessary tissue injury, and anticipating layer‐specific complications.

## 5 Procedural Equity and Skin of Color

Equity in aesthetic dermatology requires protocols that explicitly account for differential risk. PIH is more common and more persistent in darker phototypes, and pigmentary risk accumulates with repeated or overly aggressive procedures [3, 4]. A layer‐by‐layer, staged protocol can mitigate this by reducing peak inflammation, favoring conservative energy delivery, prioritizing barrier repair, and avoiding unnecessary treatment of layers that are not the dominant source of the patient's concern.

This is an important contribution of the proposed framework because procedural equity is not achieved only by lowering device settings; it also requires anatomically rational treatment selection and sequencing. Beyond technique, equity requires evidence: future trials should report outcomes by phototype, include objective pigment metrics, and evaluate whether staged protocols reduce PIH without compromising efficacy.

## 6 Outcome Assessment and Research Priorities

To make layered rejuvenation reproducible, shared language and measurable endpoints are needed. “Skin quality” can be operationalized into domains such as texture or roughness, hydration, firmness or elastic recoil, radiance, pigment homogeneity, laxity, and contour. These domains should then be linked to plausible anatomical substrates rather than treated as interchangeable aesthetic descriptors. Objective tools can complement patient‐reported outcomes: high‐frequency ultrasound may quantify dermal thickness and tissue‐plane changes, elastometry may evaluate stiffness and recoil, and colorimetry may quantify pigment change.

This measurement strategy is important because it creates a pathway for testing the multidermal framework rather than leaving it as a purely conceptual model. Future studies should evaluate whether layer‐matched treatment planning improves outcomes, safety, durability, patient satisfaction, and procedural equity compared with conventional modality‐based approaches. Prospective studies comparing layered protocols with single‐modality regimens would clarify how improvements at each depth contribute to global rejuvenation and could inform standardized, reproducible, and equitable treatment algorithms.

## 7 Discussion

Facial skin rejuvenation is inherently multidimensional, reflecting structural and functional changes that occur across multiple anatomical layers. In clinical practice, however, treatment planning is often organized around individual modalities rather than the biological substrates underlying visible skin changes. This modality‐centered approach may lead to overtreatment of certain layers while leaving the primary anatomical driver insufficiently addressed. The multidermal framework proposed in this article aims to reorient treatment planning toward a depth‐based anatomical rationale, in which specific skin‐quality concerns are systematically mapped to the tissue layers most responsible for those changes [17, 18].

The central importance of this narrative is that it provides a structured language for connecting visible skin‐quality concerns with their anatomical origins. The central novelty is that the framework does not classify rejuvenation by product category or device type, but by the tissue layer and functional target most relevant to the patient's concern.

A key implication of this approach is the potential for more rational sequencing of multimodal interventions. By recognizing that epidermal barrier integrity, dermal matrix remodeling, and subdermal structural support represent distinct but interrelated components of skin aging, clinicians may design staged treatment protocols that align with wound‐healing biology. Addressing epidermal barrier function and pigment stability early in the treatment course may help reduce the inflammatory burden of subsequent procedures, while deeper dermal remodeling or subdermal support may be reserved for later stages when tissue tolerance and treatment objectives permit. Such sequencing may improve both treatment efficiency and patient tolerability.

Another important aspect of the framework is its relevance to procedural safety. Many complications associated with aesthetic procedures arise when treatments are applied without adequate consideration of anatomical depth or regional vascular anatomy. Depth‐matched intervention selection may reduce unnecessary tissue injury and minimize exposure to higher‐risk procedural planes. Advances in high‐frequency ultrasound have further expanded the clinician's ability to evaluate tissue planes, identify previously injected materials, and assess complications in real time. As imaging technologies become more integrated into aesthetic practice, depth‐based treatment planning may increasingly incorporate ultrasound‐guided assessment and procedural decision‐making.

Thus, the framework has practical relevance while remaining conceptually distinct from a procedural manual: it explains why anatomical depth should guide treatment selection before device settings or injection technique are considered.

This framework may also contribute to more equitable aesthetic care. Patients with darker phototypes are at increased risk of postinflammatory hyperpigmentation following inflammatory or energy‐based procedures [3]. A staged, inflammation‐aware treatment strategy may reduce cumulative inflammatory burden and allow clinicians to adopt more conservative parameter selection in higher‐risk populations. Future studies evaluating layered rejuvenation protocols should therefore include phototype‐stratified outcome reporting and objective pigment measurements to better understand procedural risk across diverse patient populations.

Despite these potential advantages, several limitations should be acknowledged. The multidermal framework presented here is conceptual and is derived from established anatomical principles, published literature, and clinical reasoning rather than prospective comparative trials. Treatment responses may vary according to device technology, injection technique, patient‐specific biology, phototype, prior procedures, and environmental factors such as ultraviolet exposure. In addition, definitions and classifications of treatments such as “skin boosters” remain heterogeneous across regions and manufacturers, which may complicate direct comparisons between studies [9]. Therefore, this framework should be interpreted as an anatomy‐guided model for treatment planning and hypothesis generation rather than as a validated clinical algorithm. These factors highlight the need for standardized terminology and outcome measures in skin‐quality research.

Future investigations may help refine and validate the multidermal framework through objective measurement of treatment effects at different anatomical depths. Imaging modalities such as high‐frequency ultrasound may allow quantification of dermal thickness and structural changes following intervention, while elastometry and colorimetry may provide complementary assessment of skin elasticity and pigment homogeneity. Prospective studies comparing layer‐matched protocols with conventional modality‐based treatment strategies may clarify whether depth‐specific sequencing improves clinical outcomes, safety profiles, durability, patient satisfaction, or equity across phototypes.

As aesthetic dermatology increasingly adopts evidence‐based and individualized treatment planning, anatomically informed frameworks may provide a useful foundation for more standardized and reproducible rejuvenation strategies. The proposed framework should therefore be viewed not only as a practical planning tool but also as a research model for testing how changes at different anatomical depths contribute to global facial skin quality optimization.

## 8 Conclusion

Facial skin aging is a depth‐dependent process involving structural and functional changes across the epidermis, papillary dermis, reticular dermis, and subdermal tissues. The importance of the proposed anatomy‐guided multidermal framework lies in shifting facial skin‐quality optimization from a product‐centered or modality‐centered approach toward a layer‐specific anatomical rationale. The novelty of the framework is its organization of treatment planning according to tissue depth, functional target, and clinical skin‐quality domain rather than by device or injectable category alone.

By mapping patient concerns to their dominant anatomical substrates and sequencing interventions accordingly, clinicians may construct multimodal treatment strategies that are biologically coherent, risk‐adapted, and individualized. Attention to procedural sequencing, vascular anatomy, pigmentary risk, and imaging support such as high‐frequency ultrasound may also contribute to safer and more equitable treatment planning. Future prospective studies are needed to validate whether layer‐matched multidermal protocols improve clinical outcomes, durability, safety, patient satisfaction, and reproducibility compared with conventional modality‐based approaches.

## Author Contributions

All authors have reviewed and approved the article for submission. Conceptualization: Kyu‐Ho Yi, Suyeon Lee, Ting Song Lim, and Diala Haykal. Writing – original draft preparation: Kyu‐Ho Yi, Ting Song Lim, and Diala Haykal. Writing – review and editing: Kyu‐Ho Yi, Suyeon Lee, Ting Song Lim, and Diala Haykal. Visualization: Kyu‐Ho Yi, Suyeon Lee, Ting Song Lim, and Diala Haykal. Supervision: Kyu‐Ho Yi.

## Funding

The authors have nothing to report.

## Ethics Statement

The authors have nothing to report.

## Consent

The authors have nothing to report.

## Conflicts of Interest

The authors declare no conflicts of interest.

## Data Availability

Data sharing is not applicable to this article as no new datasets were generated or analyzed.
